# Follow-up of iatrogenic aorto-coronary "Dunning" dissections by cardiac computed tomography imaging

**DOI:** 10.1186/s12880-017-0227-3

**Published:** 2017-12-21

**Authors:** Stefan Baumann, Michael Behnes, Benjamin Sartorius, Tobias Becher, Ibrahim El-Battrawy, Christian Fastner, Uzair Ansari, Dirk Loßnitzer, Kambis Mashayekhi, Thomas Henzler, Stefan O. Schoenberg, Martin Borggrefe, Ibrahim Akin

**Affiliations:** 1 First Department of Medicine, University Medical Centre Mannheim, Theodor-Kutzer-Ufer 1-3, 68167 Mannheim, Germany; 2 Division of Cardiology and Angiology II, University Heart Center Freiburg-Bad Krozingen, Bad Krozingen, Germany; 3 Institute of Clinical Radiology and Nuclear Medicine, University Medical Center Mannheim, Medical Faculty Mannheim, Heidelberg University, Mannheim, Germany

**Keywords:** Aortocoronary dissection, Coronary computed tomography, Complication, Dunning, Percutaneous coronary intervention

## Abstract

**Background:**

Iatrogenic aorto-coronary dissections following percutaneous coronary interventions (PCI) represent a rare but potentially life threatening complication. This restrospective and observational study aims to describe our in-house experience for timely diagnostics and therapy including cardiovascular imaging to follow-up securely high-risk patients with Dunning dissections.

**Methods:**

Dunning dissections (DD) occurred during clinical routine PCIs, which were indicated according to current ESC guidelines. Diagnostic assessment, treatment and follow-up were based on coronary angiography with PCI or conservative treatment and cardiac computed tomography (cCTA) imaging.

**Results:**

A total of eight patients with iatrogenic DD were included. Median age was 69 years (IQR 65.8–74.5). Patients revealed a coronary multi-vessel-disease in 75% with a median SYNTAX-II-score of 35.3 (IQR 30.2–41.2). The most common type of DD was type III (50%), followed by type I (38%) and type II (13%). In most patients (88%) the DD involved the right coronary arterial ostium. 63% were treated by PCI, the remaining patients were treated conservatively. 88% of patients received at least one cCTA within 2 days, 50% were additionally followed-up by cCTA within a median of 6 months (range: 4–8 months) without any residual.

**Conclusion:**

Independently of the type of DD (I-III) it was demonstrated that cCTA represents a valuable imaging modality for detection and follow-up of patients with DDs.

## Background

Iatrogenic aorto-coronary dissections following percutaneous coronary interventions (PCI) represent rare but potentially life threatening complications [1]. The overall incidence is estimated around 0.02% and more common in patients with acute myocardial infarction compared to elective PCI [2]. Dunning et al. classified these dissections into three groups, where the local involvement of the ipsilateral cusp was defined as class I, the extension along the ascending aorta of less than 40 mm as class II and more than 40 mm as class III [2]. Patients with limited aortic involvement (class I and II) were described to be managed successfully by a “wait-and-see” strategy or PCI with implantation of a stent at the coronary dissection entry [2]. In contrast, Dunning dissections (DD) of type III require treatment by cardiovascular surgery [2]. While surgical treatment is still under debate, it is still under ongoing debate whether PCI with implantation of drug eluting stents (DES) at the aorto-ostial dissection might represent a valuable treatment alternative even in extensive dissections [3]. Nonetheless, large-scaled randomized controlled studies evaluating the best treatment choice in patients with DD are not available and the current scientific knowledge is limited to single case series [2].

Because patients suffering from DD underlie an increased risk of death, they may ultimately profit from an accurate diagnostic follow-up to guarantee best medical treatment [4]. However, no cCTA imaging algorithm for follow-up was yet developed in order to ensure an accurate treatment of this rare disease entity. Therefore, this study aims to describe our institutional experience to follow-up these high-risk patients.

## Methods

### Study population and patient recruitment

**Fig. 1:**
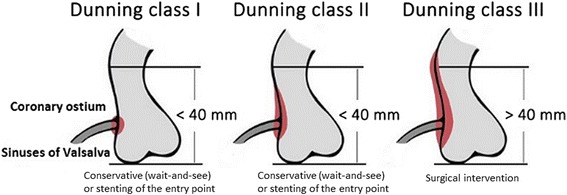
Classification of aortocoronary dissection based upon the extent of aortic involvement according to Dunning et al. [2] Class I (left), involves only the coronary cusp. Class II (﻿middle), extends up the aortic wall, but remains under 40 mm. Class III (right), contrast media extends over 40 mm up to the aortic wall

This monocentric, retrospective and observational study evaluates diagnostic and therapeutic regimens including imaging examples of patients developing iatrogenic aorto-coronary DD during clinical routine care. Identification of the patients was performed retrospectively by reviewing our in-hospital electronic documentation system, while t﻿he initial diagnostic assessment was always performed within coronary angiography at index PCI. The classification of DD was described recently by Dunning et al. [2] **(**Fig. 1 **)**.

A total of eight patients with iatrogenic aorto-coronary DDs occurring during PCI were included during routine clinical care. PCI was indicated according to current European guidelines [5] and were performed at the First Department of Medicine, University Medical Centre Mannheim (UMM), Germany, in between October 2013 and September 2016. The study was carried out according to the principles of the declaration of Helsinki and was approved by the local ethics committee of the University Medical Centre Mannheim (ethical approval number 2016-864R-MA). All participants provided verbal informed consent.

### Coronary computed tomography angiography (cCTA)

All cCTA examinations were performed by using dual-source CT scanners with a minimum of 64 detector rows (Somatom Definition or Force, Siemens Healthcare Sector, Forchheim, Germany). Our standardized imaging protocol included a single phase ECG-gated 100 kV CTA study (320 reference mAs, 0.6 mm slice collimation, rotation speed 330 ms) from the thoracic inlet to the inguinal region. Vessel enhancement was achieved via injection of 90 mL iodinated contrast medium (Iomeprol 400, Bracco, Italy) with a flow rate of 5 cm^3^/s. Automatic tube current modulation in x, y and z-direction as well as ECG-dependent tube current modulation (20–80% RR-interval) was used in all patients. All CT data acquisitions were acquired during an inspiratory breath-hold. Analysis of the cCTA images were performed on a separate workstation with a predefined high resolution screen.

### PCI of aorto-coronary dissections

Type of treatment of DD was performed according to the operator’s discretion and considered either conservatively with a “wait-and-see” strategy, interventional with PCI and sealing of the coronary dissection entry or surgical treatment.

A conservative treatment of a DD was chosen as an adequate treatment option only, when imaging results by coronary angiography or cCTA revealed limited or no further progress of the DD after the index procedure. Additionally, hemodynamic stability was required in conservatively treated patients.

PCI of DD aimed to seal appropriately the entry of the aorto-coronary dissection by implantation of DES. PCI was usually performed immediately within the index coronary angiography. An overlap of up to 3–5 mm into the ascending aorta was regarded as being sufficient to guarantee adequate sealing. An interdisciplinary discussion of all cases within a heart team was performed in all cases [5].

## Results

### Study population

A total of eight patients with iatrogenic aorto-coronary DD were included. At our institution, we have performed 3600 PCI in the study period, resulting to an incidence of 0.22%. Baseline characteristics are shown in Table 1 **.** The median age of patients was 69.0 years (IQR 65.8–74.5), and 50% were of male gender. Most patients revealed an increased cardiovascular risk profile (up to 88%) with at least one cardiovascular risk factor **(**Table 1 **).** The presence of coronary multi-vessel-disease was present in 75%. Six patients (75%) had underwent previous coronary angiography, whereas only one patient had a history of coronary artery bypass grafting (CABG). A history of heart failure was present in 38% with a median left ventricular ejection fraction (LVEF) of 60% (IQR 48.8–60.0). The median SYNTAX-II-score was 35.2 (30.2–41.2) **(**Table 1 **).**

**Table 1:** Baseline characteristics of patients with Dunning dissections. *IQR* interquartile range

Age, median (IQR)	69.0 (65.8–74.5)
Male gender, n (%)	4 (50)
Height (cm), median (IQR)	164.0 (160.0–169.5)
Weight (kg), median (IQR)	72.5 (70.0–78.0)
Body mass index (kg/m^2^), median (IQR)	27.3 (26.8–29.3)
Cardiovascular risk factors, n (%)	
Diabetes mellitus	4 (50)
Arterial hypertension	7 (88)
Smoking	5 (63)
Dyslipidaemia	4 (50)
Prior medical history, n (%)	
Coronary artery disease	
1-vessel	1 (13)
2-vessel	1 (13)
3-vessel	5 (63)
Myocardial infarction	3 (38)
Bypass surgery	1 (13)
Percutaneous coronary intervention	6 (75)
Heart failure	3 (38)
Chronic kidney disease	0 (0)
Stroke	2 (25)
Chronic obstructive pulmonary disease	0 (0)
LVEF (%), median (IQR)	60.0 (48.8–60.0)
SYNTAX-II-Score, median (IQR)	35.3 (30.2–41.2)
Laboratory values, median (IQR)	
Creatinine (mg/dl)	1.2 (0.9–1.3)
Glomerular filtration rate (ml/min)	60.0 (57.5–60.0)
Haemoglobin (g/dl)	12.3 (11.2–13.4)
International Normalized Ratio	1.0 (0.9–1.5)
Antithrombotic medication, n (%)	
Acetylsalicylacid	8 (100)
Clopidogrel	5 (63)
Prasugrel	2 (25)
Vitamin K antagonists	2 (25)

### Procedural data of patients with DD

As shown in Table 2, the most common type of DD was of type III (50%), followed by type I (38%) and type II (13%). DD occurred mostly during PCI of the right coronary artery (RCA) (88%), followed by the left anterior descending (LAD) and left main trunk (LMT) (13%). Arterial access-site was mostly of 6 French (88%) using femoral arterial access (75%) with a median implanted stent length of 30.5 mm (IQR 24.8–59.5). A mother in child catheter was used in 4 of 8 patients (Guideliner). The total procedural length was 80.5 min (IQR 63.5–108.3) accompanied by a total radiation exposure of 62 Gycm^2^ (IQR 42.3–177.3) and contrast volume of 255 ml (IQR 158.5–341.3). The median dose length product (DLP) of cCTA examinations was 113.2 mGy × cm (IQR 121.0–142.3), which corresponds to an estimated median radiation dose of 1.9 mSv (IQR 1.7–2.0).

**Table 2:** Procedural data of patients with Dunning dissections treated by percutaneous coronary intervention. ^a^elective CTO with puncture of two vessels. *CTO* chronic total occlusion, *F* french, *SD* standard deviation

Dunning dissections**,** n (%)	
Type I	3 (38)
Type II	1 (13)
Type III	4 (50)
Revascularized vessel, n (%)	
Left main trunk (LMT) and Left anterior descending (LAD)	1 (13)
Right coronary artery (RCA)	7 (88)
Multivessel disease	8 (100)
Chronic total occlusion CTO)	3 (38)
Arterial access, n (%)	
6 F	7 (88)
7 F	1 (13)
Radial	1 (13)
Brachial^a^	2 (25)
Femoral^a^	6 (75)
Antithrombotic treatment, n (%)	
Acetylsalicylacid	8 (100)
Heparin	8 (100)
Clopidogrel	6 (75)
Prasugrel	2 (25)
Abciximab	2 (25)
Vitamin K antagonists	2 (25)
Non compliant (﻿NC) balloon, n (%)	3 (38)
Procedural data, median (IQR)	
Maximum dilation pressure (atm)	18.0 (17.0–20.0)
Maximum balloon diameter (mm)	3.0 (3.0–3.5)
Maximum balloon length (mm)	15.0 (15.0–16.3)
Total stent length (mm)	30.5 (24.8–59.5)
Coronary angiography data, median (IQR)	
Procedure time (min)	80.5 (63.5–108.3)
Total fluoroscopy time (min)	19.4 (12.6–30.3)
Total contrast volume (ml)	255.0 (158.5–341.3)
Total radiation exposure (Gycm^2^)	62.0 (42.3–177.3)
CT-data, median (IQR)	
Radiation-Dose (mSV)	1.9 (1.7–2.0)
Dose length product (mGy x cm)	133.2 (121.0–142.3)
Total contrast volume (ml)	90 (90.0–90.0)

### Analysis of individual patients´ data sets

Usually patients presented with symptoms of acute chest pain during DD complicating PCI. As outlined in Table 3 **,** index PCI were mostly performed using at least 6 French (F) arterial access sheaths, either using femoral access (75%) compared to radial/brachial access (38%). PCI were performed in most cases as an elective complex PCI of chronic total occlusions (CTO) (38%) or PCI with rotablation of heavily calcified lesions (13%) as shown in Table 3. DD mostly occurred as a consequence of a too deep intubation of coronary arteries with the guiding catheter (63%), and rarely during sub-intimal wire tracking during wire externalization of a CTO. The most commonly used type of guiding catheter was an Amplatz left ﻿(AL 1) (50%).

**Table 3:** Data sets of patients with Dunning dissections. *HS* hockey stick﻿, *EBU* extra back-up, *AL* Amplatz l﻿eft, *RCB* right coronary bypass, *AR* Amplatz right, *B﻿MW* balance middleweight

Patient	Type of Dunning dissection	Access	Catheter type	Guidewire	Syntax-II-score	Procedure	Dissection-associated procedure	Type of treatment	Number and type of implanted stents	CTA post intervention time intervall (days)	Follow-Up CTA (Time interval in months)
1	III	Femoral, 7F	HS	Mailman	30.5	RCA, elective rotablation, CTO	Intubation/Engagement Guiding catheter	Ostial PCI with sealing	3 (Xience Pro 2.25/18; 2.25/15; 2.5/15 mm)	1, 2, 5	8
2	I	Femoral, 6F	EBU	BMW	29.4	LMT/LAD, elective PCI, CCS class III	Engagement of guiding catheter in ostial LMT stenosis	Conservative, wait and see	2 [Xience Pro 2.75/15; 4.0/12 mm]	x	8
3	III	Brachial, 6F	AL I	Fielder XT	36.4	RCA, elective PCI, CCS class III	Intubation/Engagement Guiding catheter	Ostial PCI with sealing	1 [Integrity 3.5/12 mm]	2	X
4	I	Femoral, 6F	RCB	PT2	34.1	RCA, elective PCI, CCS class III	Inadequate PCI of the ostial RCA without aortic overlap	Ostial PCI with sealing	1 [Integrity 3.5/18 mm]	<24 h	X
5	III	Femoral, 6F	AL 1	Pilot 50	51.7	Cardiogenic shock, complete revascularisation of RCA-CTO and LMT/RIVA/CX in one session	Unknown, postinterventional pericardial hemorrhage (1550 ml)	Conservative, protamin	6 [Xience Pro 2.75/18; 3.0/23; 2.75/23; 3.0/23; 3.5/18; 2.75/18 mm)	<24 h, 1	X
6	II	Femoral, 6F	AL 1	Whisper	37.7	RCA, elective PCI, CCS class III	Intubation/Engagement Guiding catheter	Failed rewiring, Conservative, wait and see	2 [Resolute 2.5/28 mm, Promus 3.0/20 mm]	<24 h, 1, 5, 9	X
7	III	Radial 6F	AR 2	Whisper	52.6	LMT, LAD, RCA Unstable angina	Intubation/Engagement Guiding catheter	Ostial PCI with sealing	2 [Promus 3.0/12 mm, Graftmaster 3.5/16 mm]	1, 2	6
8	I	Brachial, 6F Femoral, 6F	AL 1	Whisper	20.2	RCA, elective CTO, CCS class III	Retrograde subintimal wire tracking externalization	Ostial PCI with sealing	4 [Promus 2.75/20; 3.0/38, 3.5/20, 4.0/16 mm]	1	4

*HS* hockey stick; *EBU* extra back-up; *AL* Amplatz left; *RCB* right coronary bypass; *AR* Amplatz right; *BMW* balance middleweight

### Diagnostics and therapy treatment of DDs

Table 3 outlines data sets of cases with DD, specifically regarding target vessel and treatment by PCI. 63% of patients were treated by PCI, whereas 38% of patients were treated conservatively. Initial diagnostic assessment was visualized during index coronary angiography in 88% of the patients, while in one case the DD was detected only by cCTA. Interventional treatment by PCI was performed according to the following steps: positioning of 2nd or 3rd generation DES with an intra-aortal overlap and subsequent high pressure PCI/DES implantation. However, the respective treatment of the DD was at the discretion of the responsible operator and based on an individual decision, in consideration of the patient condition.

Follow-up CTA was not performed at predefined fixed intervals; nonetheless, the majority of the patients (88%) received at least one cCTA within the first two days after index event and 50% were additionally followed-up by cCTA within a median of 6 months (range: 4–8 months). Fifty percent of patients did not present back to our clinic, because of subjective improvement of symptoms and stable cardiopulmonary status, and therefore clinical re-assessment by cCTA was not necessary in these patients, as being decided by clinicians during routine clinical care. The follow-up of patients as well as the treatment of DD patients totally relied on the physicians being involved in clinical routine care. Their decisions were based on clinical considerations implementing a stable course with until complete healing without further re-evaluation by cCTA. In case of more severe stages of DD and as well-being based on clinical decision-making cCTA was planned and re-investigated during follow-up.

### Imaging examples according to the different types of DD

**Fig. 2:**
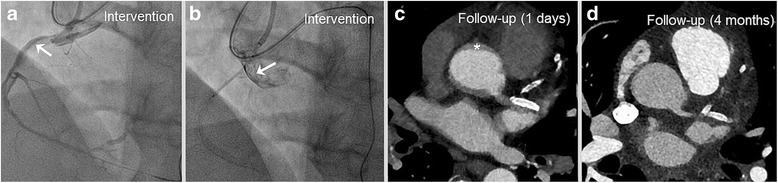
Dunning Dissection type I. Patient with CCS III and elective CTO of the RCA resulting in aortocoronary dissection. Illustration before **(a)** and after aorto-ostial sealing **(b)**, **c** The cCTA the following day showed a slight intramural hematoma with **d** complete recovery after 4 months

As shown in Fig. 2, this DD type I occurred in a patient with progredient typical angina (Canadian cardiovascular society (CCS) class III), who was planned for an elective PCI of a chronic total occlusion (CTO) of the RCA. During guiding catheter (AL 1) intubation and contrast injection of the RCA a local DD occurred at the RCA ostium **(**Fig. 2a **)**. Figure. 2b shows positioning of the DES (Promus 4.0/16 mm) overlapping into the aorta in order to seal the entry. One day after index PCI, cCTA revealed a circular intramural hematoma **(**Fig. 2c. cCTA at 4 months of follow-up demonstrates complete regression of DD without any residual hematoma as well as adequate positioning of the DES **(**Fig. 2d).

**Fig. 3:**
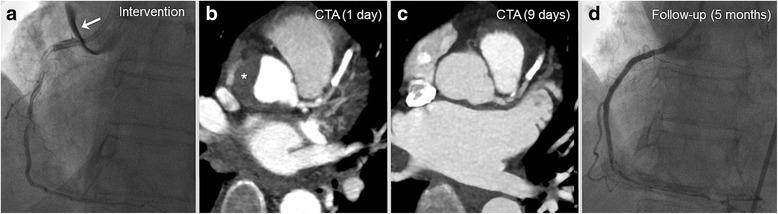
Dunning Dissection type II. Patient with 3-vessel coronary artery disease and PCI of the LAD and 90% long-segment stenoses of the RCA. **a** After intubation of the RCA with an AL1 a spiral winded DD occurred with failed rewiring. **b** cCTA confirmed the extensive haematoma. **c** The patient underwent a conservative wait and see with closed meshed cCTA with four cCTA within 9 days and **d** final satisfactory result. cCTA, coronary CT angiography; PCI, percutaneous coronary intervention; RCA; right coronary artery

Figure 3 illustrates follow-up of a patient with multi-vessel coronary artery disease and initially successful PCI of the LAD and posterlateral branch of the circumflex . After switch to the RCA and intubation with an AL1 the vessel showed a spiral winded DD II **(**Fig. 3a **)**. The instantly performed axial contrast-enhanced cCTA confirmed the extensive intramural hematoma **(**Fig. 3b **).** Due to failed re-wiring the patient underwent a conservative wait and see approach with closed meshed cCTA with four consecutive cCTA (<24 h, day 1, 5 and 9) and showed final satisfactory result after 9 days (Fig. 3c **)** and control coronary angiography after 5 months **(**Fig. 3d **)**.

**Fig. 4:**
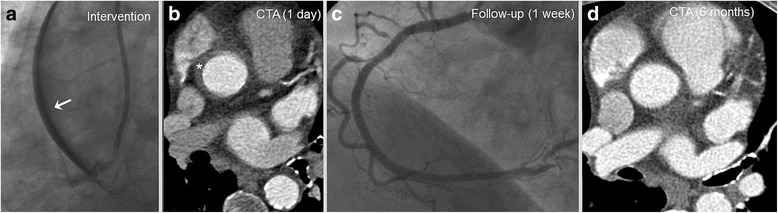
Dunning Dissection type III. Patient presenting with unstable angina in the chest pain unit. **a** Complex intubation of the RCA ostium with AR2 results in a Dunning Typ 3 dissection (arrow) with emergency sealing (Graftmaster 3.5/16 mm) without further progress of the dissection. **b** One day after the initial intervention CT confirmed an extensive dissection. **c** The invasive follow-up 9 days later showed good result, with subsequent successful re-intervention of the RCA and the LMT. **d** The follow-up cCTA confirms complete recovery after a follow-up period of 6 months. cCTA, coronary CT angiography; RCA; right coronary artery

An even more extended dissection was seen in a 76-year-old-female patient initially presenting with Non–ST-Segment Elevation Myocardial Infarction (NSTEMI) in the chest pain unit. Complex intubation of the RCA ostium with AR2 results in a very extensive DD of type 3 reaching from the ascending aorta to the brachiocephalic trunc **(**Fig. 4a **)**. Immediate PCI with implantation of a covered stent (Graftmaster 3.5/16 mm) lead to a sufficient sealing of the dissection entry without further progress. One day after the index PCI an extensive dissection was confirmed by cCTA **(**Fig. 4b **)**. The invasive follow-up 6 days later showed good result of sealing, with subsequent successful complex multi-vessel Re-PCI with implantation of multiple DES at the RCA and LMT **(**Fig. 4c **)**. The mid-term follow-up cCTA confirms complete resolution at 5 months after index PCI **(**Fig. 4d **)**.

**Fig. 5:**
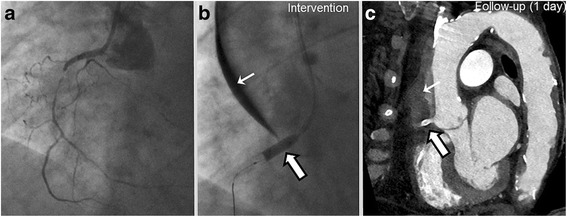
Dunning Dissection type III. **a** Angiographic findings of a patient initially admitted with CCS class II and complex coronary artery disease (SYNTAX-II-Score 36.4%) undergoing PCI of a CTO of the RCA. **b** The lesion was successfully treated with an aorto-ostial PCI/DES implantation (Integrity 3.5/12 mm) overlapping into the ascending aorta (broad arrow). **c** cCTA at day 1 confirms the extensive dissection from the RCA ostium up to the ascending aorta (slim arrow). PCI, percutaneous coronary intervention; RCA, right coronary artery

A final patient was planned for complex PCI with planned revascularization of a CTO of the RCA. After assessing coronary angiography the SYNTAX-II-Score was 36.4%. During CTO-PCI of the RCA **(**Fig. 5a **)** a DD of type III occurred during sub-intimal wire tracking with a Fielder XT CTO wire being supported by a micro catheter and final wire externalization. The lesion was successfully sealed with an aorto-ostial PCI/DES implantation (Integrity 3.5/12 mm) overlapping into the ascending aorta **(**Fig. 5b **)**. Coronal cCTA images of the ascending aorta 1 day after the index procedure confirmed extensive DD type III as well as optimal sealing of the dissection entry by the DES **(**Fig. 5c **)**.

## Discussion

The present retrospective and observational study outlines clinically relevant aspects about the timely diagnosis, treatment and imaging follow-up of patients suffering from iatrogenic aorto-coronary dissections i.e. Dunning dissection (DD) as a consequence of PCI during routine clinical care.

Modern interventional cardiology allows the chance of treating most complex and technically challenging coronary lesions by PCI [6]. The most important advantage from the patient’s perspective represents the minimally invasive treatment approach compared to open-heart surgery. As reported by Dunning et al. the rate of iatrogenic aorto-coronary dissections is higher during PCI of patients suffering from an acute myocardial infarction (0.19%) or of elective patients (0.03%) compared to conventional diagnostic coronary angiographies (<0.01%) [2]. Within the present cohort DD occurred mostly during elective and planned complex PCI and in one patient suffering from NSTEMI with subsequent cardiogenic shock. The most frequent causes of dissection occurred during interventions of the RCA (88%) and CTOs (38%). All PCIs were complex interventions being affirmed by a median SYNTAX-II-score of 35.3. After high-risk PCIs such as a PCI of a CTO close-meshed follow-up and clinical assessment is mandatory to ensure a successful outcome, because the risk of aorto-coronary dissections was markedly increased by 1.8% [7].

The vast majority occurred during PCI of the RCA, while the exact underlying pathomechanism still remains unclear [1, 8]. Within the present study the most important risk factors for Dunning dissections as being estimated by the PCI-operator contain a too deep intubation and engagement with the catheter (50%), use of an AL 1 catheter (50%), use of a mother in child catheter (50%) and subintimal wire tracking or wire externalization (13%). According to the literature, further potential risk factors seem to be the tortuous anatomy and smaller size auf the RCA, the degree of calcification mainly the aortic root, the type and manipulation of used guiding catheters, heavy coronary calcification and wires during the intervention [4, 9, 10].

Aorto-coronary dissections extending into the aortic root as described in this study were treated with central ostial PCI/DES implantation being positioned by an overlap into the ascending aorta after diagnostic confirmation by angiography. If interventional entry sealing could not perform rapidly, a conservative wait-and-see approach has a high risk of uncontrollable dissections with consecutive major vascular complications [3]. Therefore, the exact extension of the dissection is mandatory to know, while coronary angiography often underestimates the extent of the dissection due to inadequate contrast opacification. Some authors recommend intravascular ultrasound (IVUS) guided coronary stenting to ensure complete coverage of the DD and exact placement of the stent to entirely cover the ostium [11, 12]. Furthermore, only slight and few contrast should be injected carefully and directly over the guiding catheter in order not to sustain or even increase the dissection entry. IVUS or CTA may be applied instead of these direct contrast injections at coronary ostia via catheters.

The technical advances in ECG-gated CTA scanner technologies with low-radiation dose and high resolution images allow to show progression or regression of the dissection [13]. Novel technical developments allow to reduce the noise and residual artifacts with simultaneous detail preservation even in low-dose cCTA images [14, 15].

Therefore, CTA is ideally suited to confirm the presence of residual aortocoronary dissection and evaluation of its exact extent and later noninvasive follow-up. Tansie et al. investigated retrospectively eight patients with DD and described their CTA findings. Similar to our population the main side of the dissection was the ostium of the RCA (88%), but in their case series the majority of the DD occurred during conventional coronary angiography (62.5%) [16].

As DD are rare and need immediate treatment, thus randomized multicenter clinical trials are missing and even in the future will be difficult to realize. This single center experience emphasizes the use of CTAs at follow-up in order to control the exact extension of the dissection as soon as the patient is hemodynamically stable, before discharge and after a follow-up period of 3 to 6 months.

## Conclusions

DD are rare but serious complications of coronary interventions, while the extent of the dissection is defined at angiography. This study demonstrates that cCTA plays a valuable role for detection and follow-up of patients with DD. While using high-resolution scanners, cCTA can be helpful to provide precious information about the origin and extent of the dissection and is therefore a helpful tool for non-invasive follow-up.

## Abbreviations

CABG: coronary artery bypass grafting
cCTA: coronary computed tomography angiography
CTO: chronic total occlusion
DD: Dunning dissections
DES: drug eluting stents
F: French
IQR: interquartile ranges
IVUS: intravascular ultrasound
LAD: left anterior descending
LMT: left main trunk
LVEF: left ventricular ejection fraction
PCI: percutaneous coronary interventions
RCA: right coronary artery
SD: standard deviation
